# Endoluminal ultrasound versus magnetic resonance imaging in assessment of rectal cancer after neoadjuvant therapy

**DOI:** 10.1186/s12876-022-02628-9

**Published:** 2022-12-27

**Authors:** Elsayed Ghoneem, Ahmed Shekeib Abdein Shabana, Mohamed El Sherbini, Mohammad Zuhdy, Osama Eldamshety, Mohamed Gouda, Ahmed El Shamy, Gehad Ahmad Saleh, Ahmed Abdel Ghafar Saleh

**Affiliations:** 1 Department of Internal Medicine, Hepatology and Gastroenterology Unit, Specialized Medical Hospital, Faculty of Medicine, Mansoura University, Mansoura, Egypt; 2 Egyptian Liver Research Institute and Hospital, Sherbin, Mansoura, Egypt; 3 Department of Surgical Oncology, Oncology Center Mansoura University (OCMU), Faculty of Medicine, Mansoura University, Mansoura, Egypt; 4 Theodor Bilharz Research Institute, Cairo, Egypt; 5 Department of Anesthesia and Surgical Intensive Care, Faculty of Medicine, Mansoura University, Mansoura, Egypt; 6 Department of Diagnostic Radiology, Faculty of Medicine, Mansoura University, Mansoura, Egypt

**Keywords:** Colorectal cancer, EUS, Neo-adjuvant therapy

## Abstract

**Background:**

Accurate rectal tumor staging guides the choice of treatment options. EUS and MRI are the main modalities for staging.

**Aim of the work:**

To compare the performance of EUS and MRI for loco-regional staging of anorectal cancer after neo-adjuvant therapy.

**Methods:**

Seventy-three (37 male, 36 female) patients with rectal cancer after neo-adjuvant chemoradiotherapy were enrolled. Histopathological staging after surgery were used as reference for comparing the yield of loco-regional staging for EUS and MRI. EUS and MRI were done 1 month after completion of neo-adjuvant therapy.

**Results:**

Regarding post-surgical T staging, eight patients had early tumor (T2 = 16 and T1 = 9) and thirty six were locally advanced (T3 = 36), while N staging, forty patients with negative nodes and 33 were positive (N1 = 22 and N2 = 11). Comparing EUS to MRI, it showed a higher sensitivity (95.7% vs. 78.7%), specificity (84.6% vs. 68.0%) and accuracy (91.8% vs. 75.3%) for staging early and locally advanced tumor. Also, it had a higher sensitivity (78.8% vs. 69.7%), specificity (75.0% vs. 65.0%) and accuracy (76.7% vs. 67.1%) for detection of lymph nodes.

**Conclusion:**

EUS appears to be more accurate than MRI in loco-regional staging of rectal carcinoma after neo-adjuvant therapy.

## Introduction

Colorectal cancer is considered the commonest gastrointestinal (GI) malignancy. The rectal cancer represents around one third of all colorectal cancers [1]. The treatment options for rectal cancer depend mainly on the accurate tumor staging in which the plan of management changes drastically with a change in the clinical stage of the patient [2]. Superficial/early lesions (T1 or T2) without metastatic nodes can be treated with endoscopy or micro-surgery alone, whereas locally advanced/ late rectal lesions are normally treated with neo-adjuvant therapy (NAT) before surgical resection and the surgical option requires more extensive surgery with total mesorectal excision (TME). Therefore, preoperative staging is of crucial importance for adequate management [3, 4].

For rectal cancer staging, multiple modalities as magnetic resonance imaging (MRI), positron emission tomography, computerized tomography (CT), and endoscopic ultrasonography (EUS) have been used [5]. CT is superior in assessing advanced disease and presence of distant metastases, but it is not as good for assessing local staging (extent of wall invasion or presence of lymph node metastases) [6, 7].

EUS is a safe diagnostic method done though the introduction of the scope from the anal canal and rectum then visualizing the lesion, detect its morphological characters and its distance from the anal verge. EUS allows the assessment of local tumor invasion, involvement of the sphincter and lymph nodes [8, 9].

MRI is also a good tool for accurate staging of rectal cancer as it is able to assess not only wall penetration and involvement of perirectal nodes, but also presence of distant metastases and the distance between the mesorectal fascia and the tumor, which is crucial in the prediction of free circumferential margin [10, 11].

Neo-adjuvant therapy has become the standard of care for patients with locally advanced rectal cancer aiming for down staging the tumor to increase the chance of a complete resection and reduce the recurrence rate. Until recently, patients routinely proceeded to surgical resection after chemoradiotherapy (CRT), regardless of the response. Nowadays, treatment is tailored depending on the response to chemoradiotherapy. To facilitate such personalized treatment planning, there is now an increased demand for more detailed tools for response evaluation after chemoradiation [12].

The main issue in the tumor reassessment is CRT-induced changes such as inflammation and fibrosis, that make it difficult to accurately evaluate the (residual) rectal tumor and to measure response [13].

In the present study we directly compare the widely used modalities in local staging for rectal tumor, Pelvic MRI and EUS, in the same patient population with surgical pathology as the reference standard for restaging after neo-adjuvant CRT.

## Patients and methods

This is a prospective study including seventy three adult patients older than 18 years, both sexes with rectal cancer (pathologically diagnosed as adenocarcinoma before starting NAT). Included patients were affected by non-metastatic non-stenotic locally advanced rectal adenocarcinoma. Patients with metastatic rectal cancer, unfit for surgery, early stages (not requiring NAT), affected by rectal tumors other than adenocarcinoma, with previous surgical or radiation therapy or rectal stenosis were excluded. The study was performed in Mansoura University (Endoscopy Unit at Specialized medical Hospital, Oncology Center Mansoura University and Radiology department) and endoscopy Unit at Egyptian Liver Hospital during the period from May 2017 to Feb 2020. All patients were assessed by both MRI and endoluminal ultrasound before surgical management. EUS and MRI were directly compared head to head to each other in the same patient population with surgical pathology staging as the reference standard. To ensure blinding, each examination was performed by a different operator unaware of the result of the other procedure. EUS and MRI were done 1 month after completion of NAT.

Included patients should be non-metastatic operable rectal cancer received NAT for 3 months.

MRI was performed in Radiology department, Mansoura University by using a 1.5-T Signa Horizon scanner (GE Healthcare, Milwaukee, Wisc) or a 3.0-T Siemens Trio Tim scanner (Siemens, Erlangen, Germany). Patients were asked to cleanse the rectum with a water enema 2 h before the examination, and 20 mg of hyoscine butylbromide were given intravenously before beginning the examination. The MRI scans were prospectively interpreted by experienced radiology staff blinded to the endosonographic findings.

Rectal tumors, hyperintense related to muscular wall, were staged following standard criteria. Tumors confined to the rectal wall were categorized as T1–T2 lesions. Tumor signal intensity extending through the muscle layer into the perirectal fat, with obliteration of the interface between muscle and fat was defined a T3 lesion. T4 tumors were those with signal intensity extending into adjacent organs or the peritoneum (Fig. 2). Infiltrated lymph nodes were defined if they had an irregular border or mixed-signal intensity. In all studies, the mesorectal fascia was demonstrated as a low-intensity fine structure enveloping the mesorectum. All tumors (rectal tumor or infiltrated lymph nodes) located 1 mm or less from the mesorectal fascia were classified as potentially infiltrating circumferential resection margin. Tumor lesions located above the anterior peritoneal reflection had a free radial margin.

### Neo-adjuvant therapy

The treatment protocols were started immediately after diagnosis according to the stage of the disease and performance status of the patients. For bulky nodal disease or clinical T4b, neo-adjuvant therapy with FOLFOX (5-FU, leucovorin, oxaliplatin) or CAPEOX (capecitabin, oxaliplatin) was started 2–3 months prior to surgery. Neo-adjuvant radiotherapy combined with 5-FU based chemotherapy was considered for much selected patients with T4 tumor penetrating to fixed structure.

### EUS examination

EUS was performed with a linear echoendoscope (PENTAX/FUJIFILM). All the procedures were performed with the patient under conscious sedation. Briefly, patient did multiple enemas before the procedure, lying in a left lateral position during the procedure with the transducer placed in the upper third of the rectum and gradually drawn back to the anus. The normal rectal wall on EUS image has a characteristic five layers image and the tumor appears as a hypoechoic lesion. The extent of wall invasion was assessed and staged according to invaded layers. The sonographic criteria for identifying involved lymph nodes were as follows: size greater than 5 mm, hypoechoic, negative color flow doppler, sharply demarcated borders, and round shape.

### Tumor staging

For rectal cancer staging we depended on AJCC 8^th^ edition for TNM staging [14]. Endoscopically, lesion within 15 cm from the anal margin was considered as rectal cancer. Rectal cancer located within 5 cm from the anal margin was considered as low rectal tumor, 5:10 cm was considered as mid rectal tumor and 10: 15 cm considered as high rectal cancer [15].

### Statistical analysis

Data was entered and analyzed using IBM-SPSS software (version 25). Qualitative data was expressed as percentage and frequency. Quantitative data was tested initially for normality by Shapiro–Wilk’s test with data being normally distributed if *p* > 0.050. Quantitative data will be expressed as mean ± standard deviation (SD) if distributed normally, or median and interquartile range (IQR) if not. If *p* value ≤ 0.050, results were considered as statistically significant for any of the used tests.

## Results

The study enrolled 73 patients with mean age 50.47 ± 12.050 y, 37 male and 36 female. 71.2% of the tumor was in the lower zone, 27.4% were in the middle zone and 1.4% in the upper zone. On histopathological examination, 84.9% of the tumors were adenocarcinoma and 15.1% were mucoid (Table 1). Table 2 describes the EUS, MRI and post-operative pathological TN staging of the studied patients. Compared to post-operative pathological staging in all stages, the EUS staging has a sensitivity of 88.9% for the T and 77.5% for the N stage with a specificity of 96.9% for the T and 78.8% for the N stage (Table 3). On the other hand, the MRI staging has a sensitivity of 55.6% for the T and 67.5% for the N stage with a specificity of 93.2% for the T and 69.7% for the N stage (Table 4). In the early stages, comparing EUS to MRI, it showed a higher sensitivity (95.7% vs. 78.7%), specificity (84.6% vs. 68.0%) and accuracy (91.8% vs. 75.3%) for early stages. Also, it had a higher sensitivity (78.8% vs. 69.7%), specificity (75.0% vs. 65.0%) and accuracy (76.7% vs. 67.1%) for detection of lymph nodes (Tables 5 and 6).

**Table 1:** Demographic, clinical and pathological characteristics of the studied patients

	All patients (n = 73)			
	Mean ± SD	Median	Range	IQR
Age	50.47 ± 12.050	50.00	25.0, 78.0	40.50, 58.00
BMI	30.40 ± 4.901	31.00	20.0, 40.0	27.00, 35.00
*Gender*				
Male	50.7% (37)			
Female	49.3% (36)			
*Site*				
Lower	71.2% (52)			
Middle	27.4% (20)			
Upper	1.4% (1)			
*Pathology*				
Adenocarcinoma	84.9% (62)			
Mucoid	15.1% (11)			

Data is expressed as mean and standard deviation, median, range and inter-quartile range or as percentage and frequency

**Table 2:** EUS, MRI and post-operative pathological TN staging of the studied patients

	EUS	MRI	Pathology
*Early*			
T0	–	6.8% (5)	–
T1	13.7% (10)	2.7% (2)	12.3% (9)
T2	19.2% (14)	28.8% (21)	21.9% (16)
*Late*			
T3	47.9% (35)	45.2% (33)	49.3% (36)
T4	19.2% (14)	16.4% (12)	16.4% (12)
*Node*			
N0	52.1% (38)	50.7% (37)	54.8% (40)
N1	38.4% (28)	43.8% (32)	30.1% (22)
N2	9.6% (7)	5.5% (4)	15.1% (11)
*Sphincter*			
Free	93.2% (68)	93.2% (68)	–
Infiltrated	6.8% (5)	6.8% (5)	–

Data is expressed percentage and frequency

**Table 3:** Diagnostic profile of EUS staging compared to post-operative pathological staging in all stages

	T	N
Sensitivity	88.9%	77.5%
Specificity	96.9%	78.8%
PPV	80.0%	81.6%
NPV	98.4%	74.3%
Accuracy	79.5%	67.1%
Kappa	0.695	0.438

**Table 4:** Diagnostic profile of MRI staging compared to post-operative pathological staging in all stages

	T	N
Sensitivity	55.6%	67.5%
Specificity	93.2%	69.7%
PPV	0.0%	73.0%
NPV	100.0%	63.9%
Accuracy	56.2%	61.6%
Kappa	0.359	0.341

**Table 5:** Diagnostic profile of EUS compared to post-operative pathological staging in T and N stages

	T	N
Sensitivity	95.7%	78.8%
Specificity	84.6%	75.0%
PPV	91.8%	72.2%
NPV	91.7%	81.1%
Accuracy	91.8%	76.7%
Kappa	0.818	0.534

**Table 6:** Diagnostic profile of MRI compared to post-operative pathological staging in T and N stages

	T	N
Sensitivity	78.7%	69.7%
Specificity	68.0%	65.0%
PPV	82.2%	62.2%
NPV	63.0%	72.2%
Accuracy	75.3%	67.1%
Kappa	0.459	0.343

## Discussion

Rectal cancer is relatively uncommon but lethal cancer that comprise about 30% of colorectal cancer where the treatment modalities depend mainly on the stage of the disease. Various methods were used for the staging of rectal cancer before and after neo-adjuvant chemoradiotherapy including CT, Pelvic MRI, and EUS. Accurate preoperative assessment of patients with rectal cancer improves the treatment outcomes. Preoperative neo-adjuvant chemoradiotherapy has been widely accepted as the standard treatment approach for patients with intermediate risk. However, its side effects as urinary, defecatory, or sexual disorder my affect the quality of life of those patients [16, 17]. Moreover, it was found that patients who undergo TME and previously treated with radiotherapy would suffer more from these side effects like diarrhea and incontinence. This drives the European Society of Medical Oncology (ESMO) to recommend the neo-adjuvant chemoradiotherapy before surgery in cases of patients with advanced T stage only [18].

After neo-adjuvant chemoradiotherapy, both MRI and EUS offered poor diagnostic performance in the assessment of T and N stages when compared to the “gold standard”, i.e. histological examination of surgical specimens. In our study, we evaluated seventy three adult patients with rectal cancer who received neo-adjuvant therapy by EUS and MRI 1 month after completion of neo-adjuvant therapy. The mean age of our patients was 50.47 ± 12.050 y, 37 male and 36 female. When we compared EUS staging to post-operative pathological staging in all stages, we found that EUS staging has a sensitivity of 88.9% for the T and 77.5% for the N stage with a specificity of 96.9% for the T and 78.8% for the N stage. On the other hand, the MRI staging has a sensitivity of 55.6% for the T and 67.5% for the N stage with a specificity of 93.2% for the T and 69.7% for the N stage. These results were different from the results of Reginelli et al. who showed that MRI combined with diffusion weighted imaging (DWI) technique has higher sensitivity and specificity rates than conventional MRI. Specifically, the sensitivity rates were 100% for T1 and T4 stages and 91.9% for the T3 stage [19]. This difference may be due to the fact that this study was done preoperative lacking comparing results with postoperative pathology, in addition to the small number of patients in the study.

**Fig. 1:**
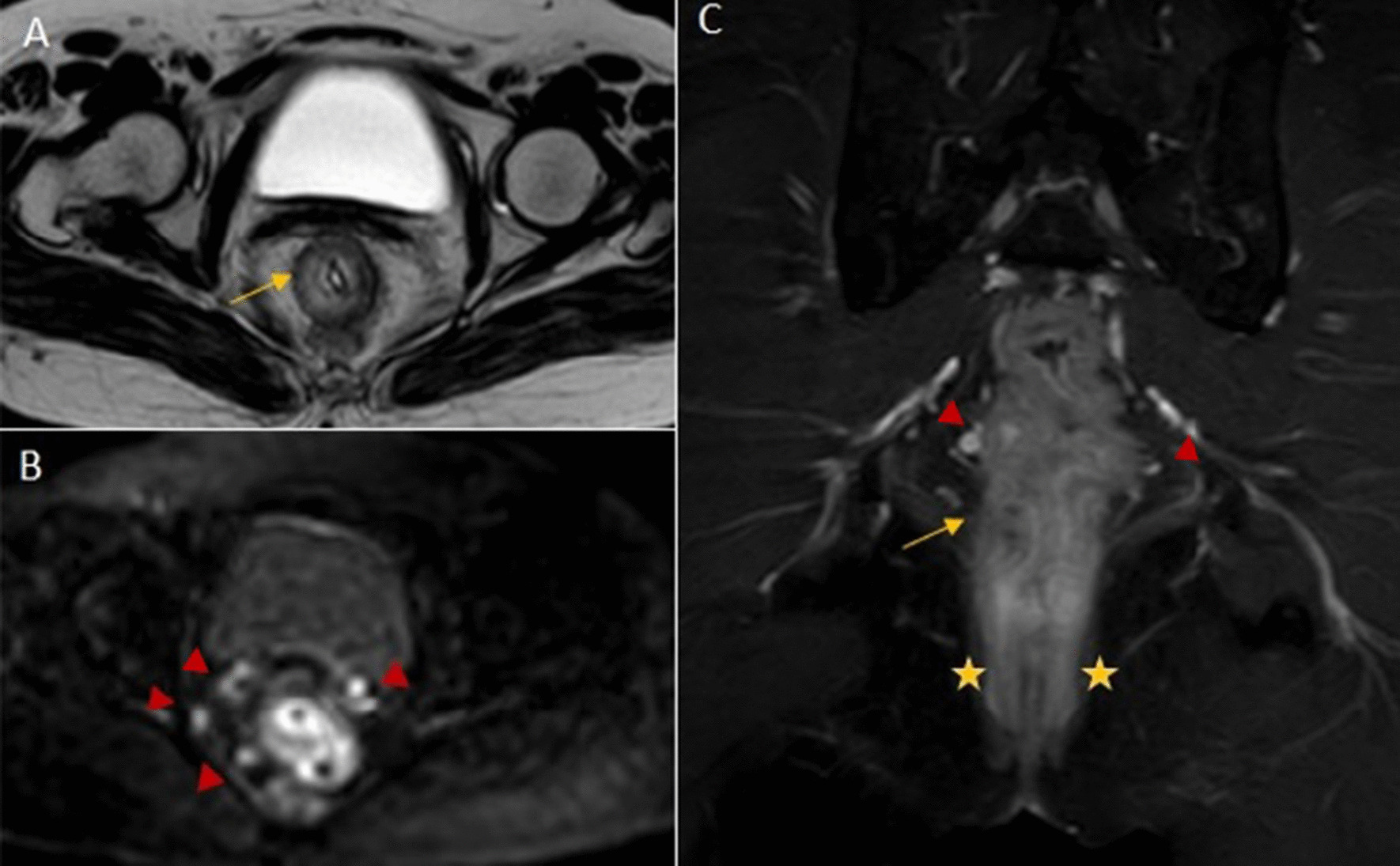
Pre and post contrast pelvic MRI: 36-year-old female post neoadjuvant rectal adenocarcinoma: Axial T2 weighted image (**A**), Axial diffusion weighted image (**B**) and coronal post contrast T1 weighted image with fat suppression (**C**): Circumferential irregular thickening of the lower third rectum (arrows) involving the external muscle layer with minimal extramural spread, no MRF involvement, extending downward into the internal anal sphincter sparing the external sphincter (asterisks). Multiple mesorectal rounded LNs (arrowheads) better detected on DW image, no extra-mesorectal LNs. MRI based staging T3, N2

**Fig. 2:**
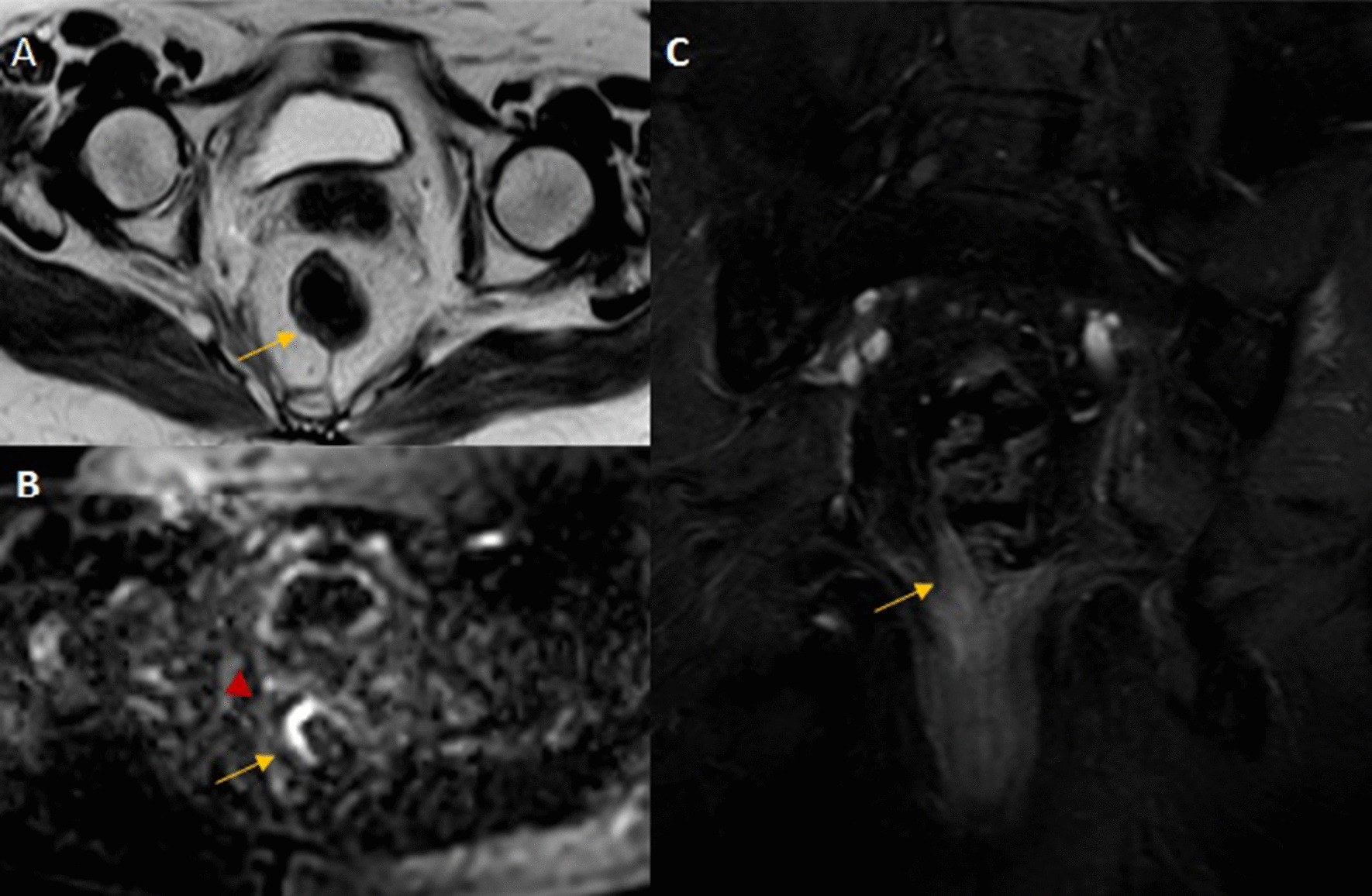
Pre and post contrast pelvic MRI: 52-year-old female post neoadjuvant rectal adenocarcinoma: Axial T2 weighted image (**A**), Axial diffusion weighted image (**B**) and coronal post contrast T1 weighted image with fat suppression (**C**): Focal irregular mural thickening of the lower third rectum from 6 to 12 o’clock (arrows) with intact external muscle layer, no extramural spread or MRF involvement, no downward extension into the anal sphincters. Tiny mesorectal rounded LN (arrowhead) better detected on DW image, no extra-mesorectal LNs. MRI based staging T2, N1

In our work, the difference between EUS and MRI staging was more pronounced in the early stages where EUS showed a higher sensitivity (95.7% vs. 78.7%), specificity (84.6% vs. 68.0%) and accuracy (91.8% vs. 75.3%). Our results are in agreement with Kav et al. who showed that the greatest difficulty in staging is the characterization of transmural tumor extension, leading to a consequent T2 over staging [20]. MRI is a valuable diagnostic tool in anal cancer staging, although the major limitation is an incorrect detection of T1 patients [21]. Several studies evaluated the MRI accuracy compared to EUS, in rectal cancer patients staging, the data suggested that EUS provides an excellent visualization of the layers of the bowel wall conversely to MRI so that EUS provides better detection of superficial tumor (Figs. 1 and 2). In evaluation of perianal and perirectal nodes, the techniques are complementary tools, while MR should be chosen for iliac and inguinal nodes [22–24].

**Fig. 3:**
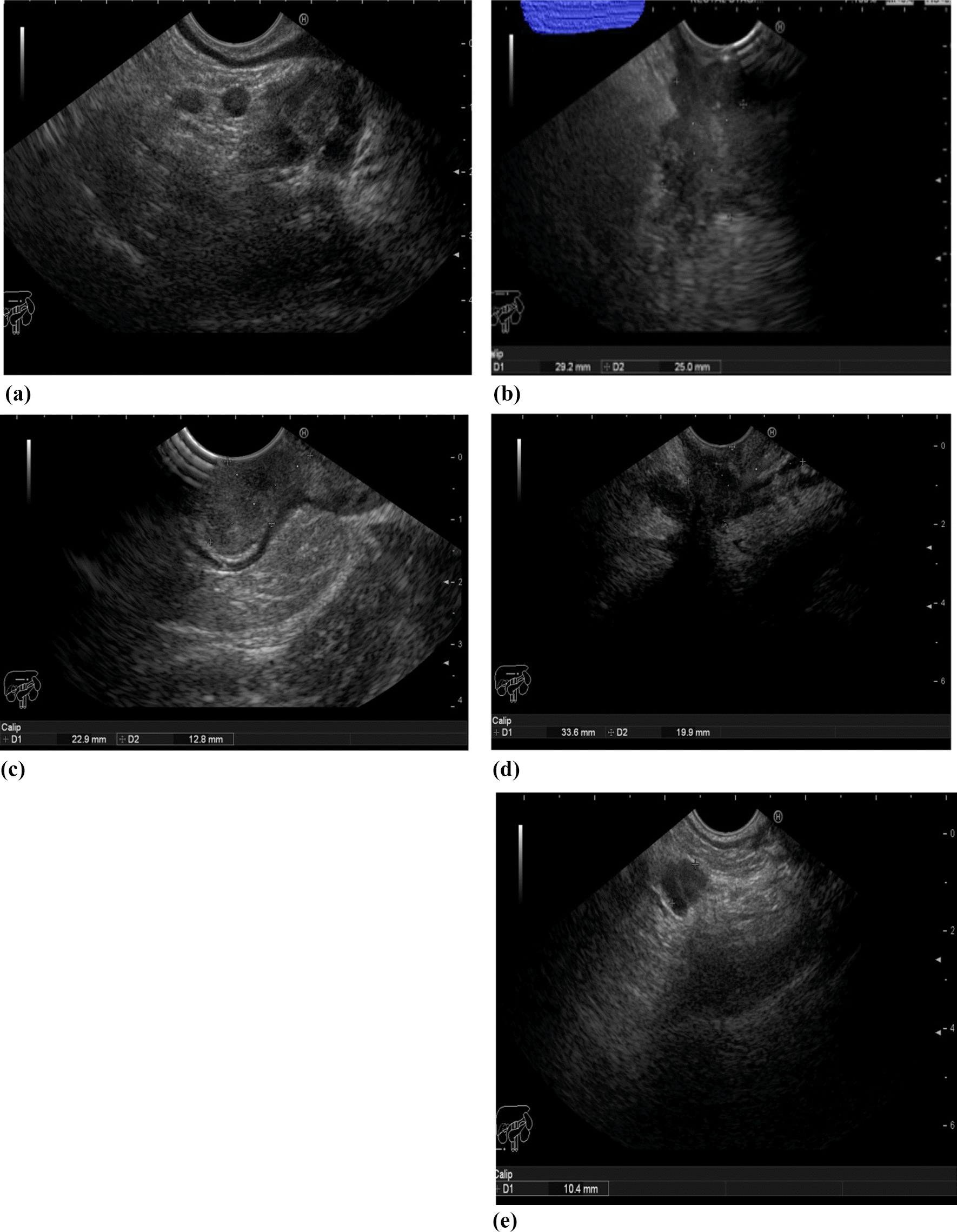
**a** EUS image of T3 rectal cancer (restaging after neoadjuvant therapy). **b** EUS image of two rounded peri rectal LNs, No hilum, in patient with rectal cancer (restaging after neoadjuvant therapy). **c** EUS image of T2 rectal cancer (restaging after neoadjuvant therapy), the lesion pass to the muscle with no serosal invasion. **d** EUS image of T4b rectal cancer with invasion to the seminal vesicles and UB (restaging after neoadjuvant therapy) **e** EUS image of rounded peri rectal LN with No hilum and irregular outline, in patient with rectal cancer (T4) (restaging after neoadjuvant therapy)

On the other hand, MRI showed lower sensitivity, specificity, and accuracy for the N stage versus the T stage when compared to postoperative pathology (69.7%, 65.0%, and 67.1% vs.78.7%, 68.0% and 75.3%). In contrast, EUS showed higher sensitivity, specificity, and accuracy for the N stage versus the T stage when compared to postoperative pathology (78.8%, 75.0% and 76.7% vs. 95.7%, 84.6% and 91.8%). From these results, EUS appears to be more accurate than MRI in the evaluation of rectal cancer patients with higher sensitivity and specificity (Fig. 3). Puli et al. reached to an opposite finding where they concluded that EUS staging of rectal cancer after neo-adjuvant chemoradiotherapy is not accurate and that MRI looks to be cost-effective in the selection of appropriate patients for neoadjuvant therapy [25]. On the other hand, endoscopic ultrasound-guided fine-needle aspiration (EUS-FNA) was proposed for N staging of rectal cancer after neoadjuvant chemoradiotherapy [26]. The contrast between our results and Puli et al. may be related to the small number of patients in Puli et al. study, in addition to, at this time rectal EUS was relatively a new technique; however the recent advances in EUS allowed more accurate evaluation.

## Conclusion

Form our results; we concluded that both EUS and MRI are accurate methods for evaluation of rectal cancer patients following neoadjuvant chemoradiation. Both techniques are complementary to each other; however, EUS seems significantly better than MRI in assessment of early stages so it is mandatory to do EUS in early stages of rectal cancer after neoadjuvant chemoradiation.

## Data Availability

All data generated or analyzed during this study are included in this published article.

## Abbreviations

GI: Gastrointestinal
TME: Total mesorectal excision
CT: Computerized tomography
MRI: Magnetic resonance imaging
EUS: Endoscopic ultrasonography
CRT: Chemoradiotherapy
NAT: Neo-adjuvant therapy
ESMO: European Society of Medical Oncology
DWI: Diffusion weighted imaging
EUS-FNA: Endoscopic ultrasound-guided fine-needle aspiration
